# Qualitative analysis of treatment needs in interstitial cystitis/bladder pain syndrome: Implications for intervention

**DOI:** 10.1080/24740527.2020.1785854

**Published:** 2020-09-01

**Authors:** Lindsey C. McKernan, Kemberlee R. Bonnet, Michael T. M. Finn, David A. Williams, Stephen Bruehl, W. Stuart Reynolds, Daniel Clauw, Roger R. Dmochowski, David G. Schlundt, Leslie J Crofford

**Affiliations:** a Department of Psychiatry & Behavioral Sciences, Vanderbilt University School of Medicine, Nashville, TN, USA; b Department of Physical Medicine & Rehabilitation, Vanderbilt University School of Medicine, Nashville, TN, USA; c Department of Psychology, Vanderbilt University, Nashville, Tennessee, USA; d Department of Anesthesiology, University of Michigan, Ann Arbor, MI, USA; e Department of Anesthesiology, Vanderbilt University School of Medicine, Nashville, TN, USA; f Department of Urologic Surgery, Vanderbilt University School of Medicine, Nashville, TN, USA; g Department of Medicine, Vanderbilt University School of Medicine, Nashville, TN, USA

**Keywords:** interstitial cystitis, painful bladder syndrome, evaluation, qualitative, sexual dysfunction, physiological, pain, chronic

## Abstract

**Background**: Interstitial cystitis/bladder pain syndrome (IC/BPS) is a debilitating condition carrying substantial psychosocial burden. Psychological treatment for IC/BPS is little studied, and there are barriers to its use in clinical management. Whether psychological treatments benefit patients with IC/BPS is unclear and we do not know whether such treatments would meet patient needs.

**Aims:** Incorporating patient-reported needs and acknowledging diversity in pain experiences can inform patient-centered interventions for IC/BPS. This project characterized the experience of living with IC/BPS and patient perceptions of needs in its treatment, with the goal of informing patient-centered treatment for IC/BPS.

**Methods:** Using both quantitative and qualitative methods, 27 females with IC/BPS participated in a focus group and completed validated self-report assessments evaluating urinary symptoms, pain, and emotional functioning. Focus groups were audio recorded and transcribed and then coded and analyzed using an iterative inductive/deductive approach. Linear regression models evaluated the relationship between psychological functioning and symptom severity.

**Results:** We conducted six focus groups between August and December 2017. Five major themes emerged from qualitative analysis: managing physical symptoms, emotional symptoms, impact on daily life and socio-contextual factors, responding to illness, and addressing needs in treatment. The physiological and emotional consequences of IC/BPS were reported, highlighting their impact on interpersonal relationships and challenges in obtaining appropriate treatment for IC/BPS. Quantitative analysis showed that depression levels were significantly associated with worsened IC/BPS symptomology, after controlling for known confounding factors.

**Conclusion:** Individuals with IC/BPS could benefit from tailored psychological interventions focusing on pain management, emotion regulation, communications skills, along with sexual dysfunction and intimacy fears.

## RÉSUMÉ

**Contexte**: La cystite interstitielle / syndrome de la vessie douloureuse (CI / SVD) est une affection débilitante qui entraine un fardeau psychosocial important. Le traitement psychologique de la CI / SVD est peu étudié. De plus, il existe certains obstacles à son utilisation dans la prise en charge clinique. Il n’est pas clair si les traitements psychologiques sont bénéfiques pour les patients et nous ne savons pas si de tels traitements répondraient à leurs besoins.

**Objectifs:** L’intégration des besoins exprimés par les patients et la reconnaissance de la diversité des expériences de la douleur peuvent éclairer les interventions centrées sur le patient pour la CI / SVD. Ce projet a caractérisé la vie avec la CI / SVD et les perceptions qu’a le patient de ses besoins pendant son traitement, dans le but d’éclairer le traitement de la CI / SVD centré sur le patient.

**Méthodes:** En utilisant à la fois des méthodes quantitatives et qualitatives, 27 femmes atteintes de CI / SVD ont participé à un groupe de discussion et ont répondu à des questionnaires d’auto-évaluation validés portant sur les symptômes urinaires, la douleur et le fonctionnement émotionnel. Les groupes de discussion ont été enregistrés et transcrits, puis codés et analysés en utilisant une approche itérative inductive / déductive. La relation entre le fonctionnement psychologique et la gravité des symptômes a été évaluée à l’aide de modèles de régression linéaire.

**Résultats:** Nous avons organisé six groupes de discussion entre août et décembre 2017. Cinq thèmes principaux sont ressortis de l’analyse qualitative : la prise en charge des symptômes physiques, les symptômes émotionnels, les répercussions sur la vie quotidienne et les facteurs socio-contextuels, la réponse à la maladie et la réponse aux besoins de traitement. Les conséquences physiologiques et émotionnelles de la CI / SVD ont été rapportées, soulignant leur effet sur les relations interpersonnelles et les difficultés à obtenir un traitement approprié pour la CI / SVD. L’analyse quantitative a démontré que les niveaux de dépression étaient significativement associés à une aggravation de la symptomologie de la CI / SVD, après contrôle des facteurs de confusion connus.

**Conclusion:** Les personnes atteintes de CI / SVD pourraient bénéficier d’interventions psychologiques sur mesure mettant l’accent sur la prise en charge de la douleur, la régulation des émotions, les compétences en communication, ainsi que le dysfonctionnement sexuel et la peur de l’intimité.

## Introduction

Interstitial cystitis/bladder pain syndrome (IC/BPS) is a chronic and costly condition affecting up to 8 million individuals in the United States.^1^ Hallmark symptoms of IC/BPS include pain in the pelvis, urogenital floor, or genitalia; urinary urgency and frequency; and pressure in the bladder.^2,3^ A high-need, high-cost population,^4^ patients with IC/BPS are medically complex and often unresponsive to surgical intervention, with most treatments targeting only symptom control and lacking effectiveness.^5^ Psychosocial comorbidities such as anxiety, depression, suicidality, and trauma-related symptoms are prevalent in individuals with IC/BPS, and these intensify the illness.^6–9^ Specifically, a recent systematic review^10^ indicated that there is significantly increased likelihood of anxiety and depressive disorders occurring prior to and following the onset of IC/BPS. Symptoms of these conditions, including helplessness, catastrophizing, and suicidal ideation, were found to be associated with increased bladder pain severity, overall impairment, and reduced likelihood of returning to work.^6,7,10^ Further, the consequences of living with IC/BPS include sleep disturbance and fatigue, which in turn worsen daily functioning. This suggests a strong association between and reinforcement of psychological symptoms and bladder-specific symptoms in IC/BPS.

Recent research notes a lack of interdisciplinary mental health intervention in urology despite evidence of the psychological difficulties that accompany urological conditions and recommendations for their management.^11^ Both the American and Canadian Urological Associations recommend approaching IC/BPS through conservative, noninvasive treatment initially to establish symptom control and improve quality of life.^3,10^ Recommended first-line interventions include education, dietary modification, bladder training, pain management, and stress management. Although national guidelines recommend psychological interventions such as stress management as an aspect of first-line treatment for IC/BPS,^5^ these interventions are understudied and underutilized due to limitations in provider practice focus, intervention availability, expertise, and time constraints. Specifically, three preliminary investigations to date have examined the potential of psychosocial intervention for IC/BPS, using online health education, relaxation training, and one study piloting a group mindfulness-based intervention not specific to chronic pain.^12–14^ Existing studies are limited by small sample sizes and a lack of follow-up. Effective and standardized psychological interventions specifically for IC/BPS that can be disseminated to providers and patients have yet to be developed. Moreover, there is high variability in interventions studied to date, some of which are broadly directed toward enhancing self-regulation and others that provide specific health behavior education without an interventionist present. We do not know how patients would receive such interventions and whether or not they would meet the needs of the population. For example, it is unknown whether education alone is a sufficient intervention or whether patients require the presence of a therapist. Quality of life in IC/BPS is hindered by significant sexual dysfunction and pain and embarrassment and shame due to symptoms.^15–17^ Recommended psychological interventions include cognitive–behavioral self-management programs, which help patients build confidence and skills in preventing, coping with, and reducing pain; however, these interventions do not traditionally address sexual pain and dysfunction.^18^

In addition to a pressing need for psychological interventions for IC/BPS, a simultaneous call to action exists in the field of cognitive–behavioral pain management. Two major criticisms of current cognitive–behavioral approaches to pain management include the (1) lack of illness-specific interventions and (2) use of generic measurement outcomes not informed by patient need.^19^ Existing cognitive–behavioral approaches to chronic pain could be enhanced by developing condition-specific intervention strategies and measures that are sensitive to the full range of patient needs, including emotional and interpersonal concerns.

This project aimed to characterize the experience of living with IC/BPS and patient perceptions of needs in its treatment using both qualitative and quantitative analyses. We first sought to describe the physiological, cognitive and emotional, and interpersonal impacts of living with IC/BPS and patient perceived needs in the management of IC/BPS to potentially inform development of a psychosocial intervention for this condition. We then aimed to provide a conceptual framework to guide the understanding of IC/BPS. It is our hope that this information can be both a useful resource for future intervention development in this population and provide clinicians treating individuals with bladder pain and urologic symptoms with an in-depth account of unique patient experiences to inform the management of IC/BPS and associated conditions.

## Method

### Study Design and Participants

We conducted mixed methods research via focus groups and surveys of patients with IC/BPS. We identified patients in person through outpatient clinics at a large academic medical center, via a hospital-wide listserv, and online through a national clinical research participation repository (ResearchMatch^20^). Prior to study enrollment, referring medical providers or trained study personnel screened participants for study eligibility. Inclusion criteria were English-speaking adult females (age >18) with an existing diagnosis of IC/BPS. We confirmed the presence of IC/BPS via medical record review. In three instances where urologic medical records could not be accessed, we used validated cutoff scores on a urinary symptom screening instrument (described below) in addition to self-reported diagnosis to indicate the presence of IC/BPS. Exclusion criteria were the presence of conditions that could interfere with focus group participation such as cognitive or psychotic disorder listed in the medical record, current substance dependence, or acute emotional distress such as active suicidal ideation at the time of screening (e.g., if participants responded “yes” to the question “Are you currently experiencing severe emotional distress or thoughts of harming yourself?”).

### Study Procedures

The Institutional Review Board (IRB) at Vanderbilt University Medical Center reviewed and approved all study procedures (IRB Study #170653). We invited eligible participants to complete a brief series of validated questionnaires and participate in a single focus group, lasting up to 90 minutes in total. A total of six focus groups occurred from August to December 2017. Group size ranged from 2 to 12 participants. All participants provided informed written consent to participate in the project. Participants completed consenting procedures and questionnaires upon arrival (15 minutes) and then engaged in a 60- to 75-minutes group discussion. An expert in qualitative research (K.B.) facilitated groups with at least one member of study personnel present. The group discussion followed a semistructured moderator’s guide with three major domains: (1) patient experience of living with IC/BPS; (2) treatment experience and needs; and (3) desire for alternative treatment strategies to address IC/BPS symptoms. Each section included a list of prompts that could be used to facilitate discussion. The guide was developed by the coauthors in collaboration with the Vanderbilt Qualitative Research Core. The focus groups were audio recorded and transcribed using an IRB-approved transcription service (rev.com). Following study completion, individuals received a US$50 gift card.

#### Quantitative Measures Used

The quantitative measures used in this investigation were informed by national recommendations in the study of chronic pain,^21^ ongoing nationwide investigative trials into IC/BPS,^22^ and recent recommendations for comprehensive psychosocial evaluation of urologic patients.^23^ Due to the limited time available for participants, we chose to prioritize measures of pain, urinary symptoms, and affective functioning in our selection of instruments. The purpose of quantitative analyses were to contextualize qualitative themes by assessing relationships between emotional and physical symptoms. We also examined descriptive data to assess how our sample compared to those of other investigations on levels of symptom severity and psychological distress.

#### Demographics Information and Clinical Data

Patients completed an 11-item brief demographic questionnaire indicating age, race, religious orientation, and household income. Patients responded to questions about their diagnoses and treatments.

#### Pain

**Brief Pain Inventory–Short Form** (BPI)^24^: The BPI is a validated brief assessment measuring pain intensity at its least, worst, and on average in the past 24 h. The BPI also assesses pain interference in several life domains. All items are measured on a 11-point Likert scale, with higher scores indicating more severe pain (0 = *no pain*, 10 = *pain as bad as you can imagine*).

**Michigan Body Map–revised version** (MBM)^25,26^: The MBM is a self-report measure used to assess the location(s) of chronic pain complaints and widespread body pain across 35 potential pain sites. Scores are calculated through summing total pain sites endorsed, with higher scores indicating greater widespread pain (total scores ranging from 0 to 35). The MBM has acceptable test–retest reliability and face, convergent, and discriminant validity as an index of widespread pain.^27^

#### Urologic Symptoms

**The O’Leary-Sant Symptom and Problem Index** (ICSI/ICPI)^28^: The ICSI/ICPI is a validated and widely used eight-item self-report measure of urinary and pain symptoms and how problematic these symptoms are for individuals with IC/BPS. The measure assesses both symptoms and problems of IC/BPS each with four questions, yielding a symptom score (ICSI), problem score (ICPI), and total severity score. Symptom scores (ICSI) range from 0 to 21 and problem scores (ICPI) range from 0 to 16, with a total ICSI/ICPI combined score ranging from 0 to 37. All items are scored on a four-, five-, or six-item Likert scale corresponding to each symptom or problem question (e.g., 0 = *not at all* to 5 = *usually* or 0 = *no problem* to 4 = *big problem*). Total scores (ICSI > 6, ICPI > 6) greater than 12 are considered severe symptoms and have a 90% sensitivity and 95% specificity in discriminating those diagnosed with IC/BPS from symptomatic controls.^28^ Further, symptom scores (ICSI > 5) have been shown to positively screen for IC/BPS in undiagnosed individuals ultimately diagnosed with the condition, with 94% sensitivity and 50% specificity.^29^ For this study, we used the total score as an indication of symptom severity and also to confirm the presence of IC/BPS in three cases where urologic medical record information was not available using the recommended cutoff of >5.

#### Affective Vulnerability

**Patient Health Questionnaire-9** (PHQ-9)^30^: The PHQ-9 is a nine-item brief questionnaire to screen for the presence of depressive symptomology with a 4-point likert scale (0 = *not at all*, 3 = *nearly every day*). Item responses are summed, with scores ranging from 0 to 27, with >14 indicating moderate to severe depression symptom severity. It is a reliable and valid measure of depression symptom severity and commonly used in medical settings as both a clinical and research tool.^31^

**Patient-Reported Outcomes Measurement Information System (PROMIS) Anxiety Scale**^32,33^: The PROMIS Anxiety Scale consists of eight items asking about specific symptoms related to anxiety within the past week using a 5-point Likert scale (1 = *not at all*, 5 = *extremely*). Total raw scores are summed and can range from 8 to 40, which convert to standardized *t*-scores following a normal distribution (average *t*-score = 50, SD = 10), with higher scores indicating greater symptom severity. These scales have been developed for use in clinical trials and are validated across populations.

### Data Analysis

#### Qualitative Analysis

Qualitative data coding and analysis was managed by the Vanderbilt University Qualitative Research Core, led by a PhD-level psychologist (D.S.). Analysis of qualitative data was carried out using SPSS25^34^ software and Microsoft Excel. Focus group content analysis was carried out with an iterative inductive–deductive^35^ approach. We followed Consolidated Criteria for Reporting Qualitative Research guidelines,^27^ which applies a systematic evaluation to qualitative data using replicable evidence-based analysis and reporting methods. A hierarchical coding system was developed and refined using the focus group guide and a preliminary review of two transcripts. Major coding categories included (1) living with IC/BPS; (2) social/mental health support; (3) treatment experiences; (4) provider capabilities; and (5) treatment needs. These main categories were further divided into subcategories, with some subcategories having additional levels of hierarchical divisions. Definitions and rules were written for the use of each category. The coding system is detailed here: https://healthbehavior.psy.vanderbilt.edu/McKernan/CodingSystemMcKernan.pdf.

*Coding Process*: Two experienced qualitative coders first established reliability in using the coding system, resolving any discrepancies through group discussion, and then independently coded the six focus group transcripts. Coders categorized each participant statement as loading onto a specific theme (or themes) and given a descriptive label or code(s). The coded transcripts were then combined into a single document and sorted by code.^36^ The number of mentions for each theme and the number of groups for which the theme emerged were recorded as a way to organize presentation of the themes. Though the use of theme frequency data in qualitative research is not without controversy, we make use of frequency values to provide an indication of what experts referred to as the *internal generalizability* of themes to our focus groups.^37^ Therefore, in its presentation, frequency should not be interpreted as importance of a given theme to the experience of IC/BPS.

*Conceptual Framework Development*: Using the inductive–deductive^38^ approach, we developed a conceptual framework that illustrates that there are biological, psychological, and environmental circumstances that influence the lived experience of patients with IC/BPS. This approach incorporates existing theory as a general scaffolding to build upon with newly generated material from focus groups that illuminate illness-specific content to capture patient experiences of living with IC/BPS. Deductively, theoretical contributions to the analysis were informed primarily by the biopsychosocial model of pain,^39^ a widely accepted heuristic model of chronic pain, propounding that pain is a unique experience to the person: filtered through one’s history, social environment, emotional state, and physiological processes that together interact to represent one’s experience and expression of illness. Inductively, the codes and themes from the focus groups were used to fill in the details of the conceptual framework.

#### Quantitative Statistical Analysis

Analysis of quantitative data was carried out using R.^34^ We calculated means, medians, and descriptive statistics of demographics and measures of psychosocial, pain, and symptom functioning. We assessed the relationship between depressed mood (independent variable) and symptom severity (dependent variable) using multiple linear regression, accounting for covariates of age and time since diagnosis, which have both been associated with increased symptom and depression severity in previous investigations.^15^ Specifically, one previous case–control study of newly diagnosed individuals with IC/BPS (<6 months) reported higher depression symptom scores than previous investigations using chronic cases,^40,41^ and a recent large epidemiological study indicated that relative risk of depression in IC/BPS decreased with age.^11^ These analyses were descriptive, not testing any particular hypothesis. Therefore, we used an alpha = 0.05 (level of significance) for each model as an indication that there may be a significant effect. However, as descriptive analyses, any of these results should be replicated before considered at the same level of inferential evidence.

## Results

### Sample and Participant Characteristics

**Figure 1.**
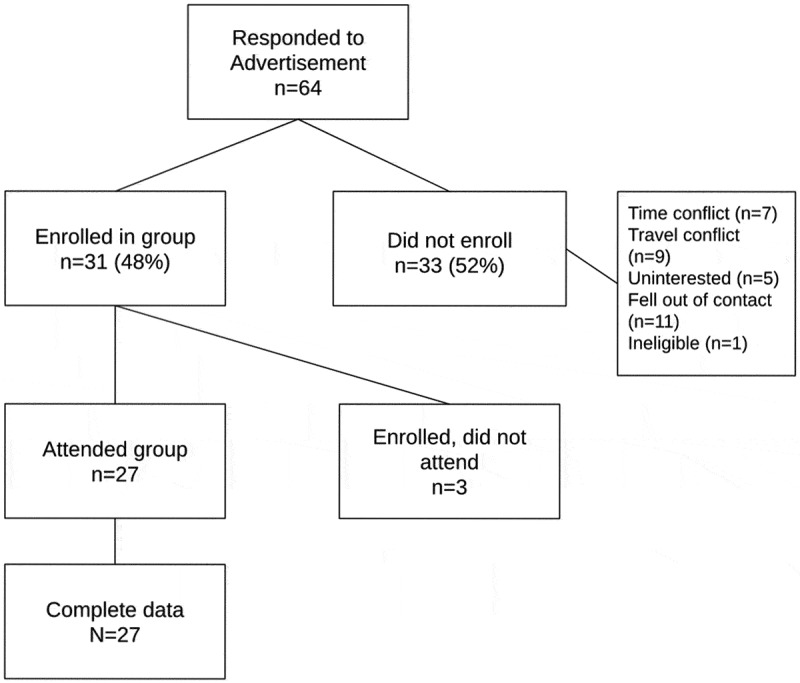
Study flow figure

Of a total of 64 potential participants who responded to the study advertisement, 31 enrolled in a focus group (48% enrollment rate) and ultimately 27 women participated in the study, representing 43% of the original eligible sample. Figure 1 indicates the study flow. The participants were recruited through a variety of sources: 37% (*n* = 10) were recruited in person; 26% (*n* = 7) through a research listserv; 22% (*n* = 6) through ResearchMatch, a national clinical research registry; and 15% (*n* = 4) from previous research who had indicated interest in further studies they may qualify for. Table 1 lists demographic and clinical data for all participants. Recruitment methods allowed for a diverse sample of participants across age, socioeconomic status, and disease duration. Particpiants were 45 years old on average (SD = 16.30), predominately White non-Hispanic (85.2%; *n* = 23), and most had a bachelor’s degree or higher education (63.0%; *n* = 17). These characteristics reflect the demographic and racial characteristics of IC/BPS reported in larger cohort studies.^12,13^

**Table 1.** Demographic and clinical characteristics of participants

	Total (*N* = 27)
Variable	Mean (SD)/count (%)
Demographic	
Age	45 (16.30)
Ethnicity	
White	24 (88.89%)
Black	2 (7.47%)
Multiracial	1 (3.7%)
Marital status	
Single (never married)	10 (37.04%)
Married or domestic partnership	10 (37.04%)
Divorced/widowed	7 (25.93%)
Receiving disability?	
Yes	2 (7.47%)
No	25 (92.59%)
Household income	
Under $10,000	1 (3.70%)
$10,000–19,999	3 (11.11%)
$20,000–$50,000	8 (25.93%)
$50,000–$100,000	11 (33.33%)
$100,000–$150,000	3 (11.11%)
$150,000 or higher	2 (3.70%)
Rather not say	*3*
Education	
High school diploma or equivalent	3 (11.11%)
Vocational/technical school	3 (11.11%)
Some college	4 (14.81%)
Bachelor’s degree	10 (37.04%)
Master’s degree	5 (18.52%)
Doctorate or professional degree	2 (7.41%)
Employment	
Employed full-time	13 (48.15%)
Employed part-time	1 (3.7%)
Unemployed	6 (22.22%)
Retired	4 (14.81%)
Unable to work	3 (11.11%)
Clinical	
Age of diagnosis	37.70 (17.27)
Age of first symptoms	30.26 (17.15)
Interstitial cystitis total severity	22.63 (8.45)
Interstitial Cystitis Symptom Inventory	12.44 (4.80)
Interstitial Cystitis Problem Inventory	10.19 (3.98)
Michigan Body Map	10.52 (11.01)
BPI intensity	4.33 (2.39)
BPI interference	4.13 (2.81)
PROMIS anxiety	20.77 (8.29); *t* = 59.4
PHQ-9	8.46 (7.27)
PHQ-9–suicidality question (>0)	14.81% (4)

BPI = Brief Pain Inventory, Short Form; PROMIS = Patient-Reported Outcomes Measurement Information System; PHQ = Patient Health Questionnaire-9.

Qualitative analyses revealed five major theme categories that together comprise the patient lived experience of IC/BPS: managing physical symptoms, emotional symptoms, impact on daily life and socio-contextual factors, response to illness, and addressing needs in treatment. Thematic saturation was reached after six groups. We will review each theme in detail, along with subthemes that emerged to provide further context to patient experiences living with IC/BPS.

#### Theme 1: Managing Physical Symptoms

(Table 2) Physical symptoms managed by participants consisted of bladder and pelvic pain, sleep difficulties, nausea due to pain, and persistent fatigue. For some, pain dominated these complaints, including persistent pain and severe dysuria (quotation 2.01). Others expressed a combination of symptoms. All groups expressed difficulty with urinary urgency and frequency, although to a varying degree. One participant described significant urgency affecting daily activities and decisions to leave the house (quotation 2.03). Another reported frequency of six to eight episodes per hour beginning as a teenager, resulting in accommodations in high school and dropping sport activities (quotation 2.04).

**Table 2.** Physical and emotional symptoms. Subthemes are sorted by number of mentions (decreasing) within each theme

Themes and subthemes	Mentions	Group mentions (out of 6)	Quotation number	Example quotation	Group
Physical					
Pain/nausea/fatigue/sleep	102	6	2.01	I have a really high pain tolerance. I am miserable right now. I feel like I have acid just pouring out of me every time I go to the bathroom. It makes you feel uncomfortable, dirty.	6
			2.02	I’ve put people to the test over the last seven years. … My daughter can tell you, she’s like, “Mommy, why are you in the bed? Mommy, why? You’re always sleeping?” It’s hard to have her understand.	5
Frequency/urgency	45	6	2.03	I know where all the bathrooms are at. At nighttime it’s not so bad ’cause I can pull over, but during the day, can’t make too many plans. Too far away or go somewhere and get stuck. Like, oh, we don’t have a bathroom. … Now I have to go behind your building.	4
			2.04	For me, I was diagnosed at 15, and I was a freshman in high school. I literally woke up one day and started going to the bathroom six to eight times every hour. I had to quit playing sports. By the time my senior year hit, I only needed two credits to graduate, so I was able to work it out where I went to school for two hours and I went home.	5
			2.05	My mom was like, “You just went to the bathroom.” I was like, “I know, but I have to go again.” “No, you don’t.” “Yes, I do.” Every time I went to the bathroom it wasn’t a lot, but it was the fact that I went.	5
Emotional					
Emotional effects on IC symptoms	49	6	2.06	Well, naturally I’m very anxious and if anything is stressing me out, if I’m having problems with my boyfriend, or I got fired from my job a few months ago because of this, that just has kept … the cycle of pain, stress, anxiety, pain, stress, anxiety. There’s not a break. Anything is stressful.	3
			2.07	I know stress is painful. Easy and simple as that. If I get stressed out at work or anything like that I can feel everything almost tighten up. And once I’m tense, there’s no going back from there. You can’t really backpedal out of it.	1
IC impact on emotional state	49	6	2.08	Anytime you have a lot of pain, that controls you. It does come on 0 to 60 in about two seconds. You can be in a wonderful mood, having a great time, the next thing you know, you’re sitting in a corner going, “Where’s the nearest bathroom?” You gotta find it now. It controls you. It controls your whole nervous system, I guess. You rejoice when it’s not there, you cry when it is. I don’t know how else to explain it. It’s very painful.	1
			2.09	The worry that I have all the time constant no matter what. … I think if you can somehow get everything working right, get your brain working right and not be on all these drugs that they keep putting you on, you maybe have a better quality of life.	6
			2.1	But I’m tired. I’m tired of this disease. I’m tired of thinking about it. I’m tired. But it takes multiple resources to get what you need and almost stabilize it to where it’s … you can tolerate it.	1
			2.11	I guess for me it’s always kind of like I have anxiety about it all the time. … I’ve had chronic bladder infections and UTI’s. I may have had this for three years but never was diagnosed until this year. They were always putting me on antibiotics. I’m just tired of that life. I don’t want to be on any antibiotics at all, ever. I think it’s ruining my body.	2
			2.12	I think it’s depressing. I find I get very blue, I get down. I get discouraged. I definitely get anxious about the bathroom thing. How long can I go in a movie until I have to interrupt the movie, everybody’s movie. I find my symptoms getting worse as I get older, and I think it’s for a variety of reasons. I just get down. I get down about myself, I get down	1
Always thinking of IC	12	5	2.13	My pain is so severe that I can’t concentrate. You just can’t. If I was in a meeting, that’s why I’m like, “Okay, I know I have to void at least once an hour.” … I’ll have a sensation of pain, and it will go from a one to about a nine in about 5 minutes or 10. A very short period of time it gets excruciating, and you’re just like, “Oh my goodness.” It’s just hard to concentrate on a conversation or a meeting where you’re making decisions and so forth.	1
			2.14	When those little moments, little victories or whatever, it’s like you get. … To me it’s like, “Oh, man. I’m gonna enjoy this until it comes back.” ’Cause you know it’s coming. It’s like a bill collector.	3
			2.15	I mean, I think more about just pretending you’re fine is I don’t want to be the girl at work that people are noticing, like you’re always leaving meetings to go to the bathroom. So things like that I feel like I’m thinking a step ahead. Or you’re in a meeting and you’re in so much pain, and I feel like I’m looking at the person talking and it’s something that always comes up to me is, “You have no idea how much pain I’m in right now, but yes, I’m gonna pretend I’m listening to you, what you’re saying.”	1

IC = interstitial cystitis; UTI = urinary tract infection.

#### Theme 2: Emotional Symptoms

(Table 2) Participants voiced pervasive and severe emotional distress related to IC/BPS. In all groups, participants acknowledged the reciprocal nature between emotional states and symptomology, with emotional distress both preceding and following symptoms. For example, participants reported distress consistently leading to pain (quotation 2.06). Another participant detailed her experience of sudden, unexpected urgency and pain rapidly altering her mood to sadness (quotation 2.08). Participants frequently described experiences of depression in reaction to symptoms recurring (quotations 2.08, 2.12) and experiencing stress related to a lack of control over symptoms and ineffective treatments (quotation 2.10).

The cognitive impact of IC/BPS was also noted, involving excessive planning, cognitive preoccupation, rumination about symptoms, and concentration difficulty due to symptoms. For example, one participant described her experience using an example of planning for a single work meeting and monitoring fluid intake, restroom schedule, bathroom locations, and fear of pain with increasing urine concentration (quotation 2.15). Another participant described difficulty enjoying symptom-free periods due to looming concern and rumination of rapid symptom return (quotation 2.14).

#### Theme 3: Impact on Daily Life and Socio-contextual Factors

(Table 3) Participants described widespread social burden and life-altering effects of IC/BPS symptoms. Social impact was discussed in all six groups, with the most frequently cited concern involving the negative effect of IC/BPS on romantic relationships and intimacy. Participants described fear and avoidance of sexual activity out of concern that sex will exacerbate symptoms (quotation 3.01) or cause pain (dyspareunia; quotation 3.02). One participant discussed not knowing how to communicate her symptoms and fears surrounding sexual activity to a potential partner (quotation 3.07). Multiple participants acknowledged not initiating dating relationships due to IC/BPS (quotation 3.02) and IC/BPS ending existing relationships (quotation 3.03).

**Table 3.** Impact on daily life and socio-contextual factors. Subthemes are sorted by number of mentions (decreasing) within each theme

Theme and subtheme	Mentions	Group mentions (out of 6)	Number	Example quotation	Group
Relationships/other interactions					
Romantic	38	6	3.01	See, what happened was I had sex with my boyfriend last year, okay? I got a UTI. Then I got a horrible flare. … That made me afraid of having sex because it’s like, “Jeez, I got that UTI and that was miserable.”	6
			3.02	Yeah, I had to quit dating, because I don’t want to try to explain that sex is painful and I don’t want to even go there. … I’m even having trouble with my own family, so how can someone you’re just dating.	3
			3.03	It ruined my marriage. It ended up in a divorce.	3
			3.04	It’s just a cycle and he’s great and he’s really supportive, but then I feel guilt that I can’t do the things. I can’t have sex with him [but] I want to have sex with him.	6
			3.05	Yeah, intercourse is far and in between. There’s one person right now that I’ve had in the last couple weeks, and I haven’t had any pain. I haven’t had any “don’t touch me” moments. I don’t know. I guess you can say this may be the one for me. I don’t know. I push people away, because for me to explain my disease in depth, it scares them.	5
			3.06	I’m in a relationship for two years. That can … I don’t know why I’m getting emotional right now. That’s just really hard because you want to be intimate with the person that you love and you can’t because you have that fear of having that terrible pain. It’s awful.	2
			3.07	Definitely the relationship aspect. I don’t even want to date anymore because that is one of my worst fears. I’ve had experiences in previous relationships. Some people were not very … They just don’t understand. How do you explain that? I can totally relate to that part of it, too. I don’t know what’s going to happen.	2
Reactions	30	6	3.08	The type of mentality that me and my mom have is like, “We need an answer.” Finally, they diagnosed me. You have this. This is very rare. It’s overwhelming. You’re going into a doctor’s office at the age of 15 every week with a bunch of old men. They’re looking at you like, “Why is she here? What is she doing here?”	5
			3.09	I did not expect to start crying. But speaking of the relationship part, you’re talking about your mom and wanting to thank her. I realized I don’t have that at all. I don’t feel like my family has gotten to a point where really anyone believes me yet. I’m really pretending around them, and they don’t understand any of the diet stuff at all. It’s very hard to go home and say, “I can’t have that.” And they’re like, “Well do we really have to get a different dinner just for you? Are you serious? You’re making it up.”	1
Family	27	5	3.1	Like I said, when I was diagnosed, I was still at home. I wasn’t married or anything like that. My dad, literally, he just thought I was crazy. It was, “You’re having a psychotic disorder.” I think the only saving grace that I had was that I still lived at home, and so he’d take me to my appointments. Like I said, my doctor just looked at them and said, “This is real.”	1
How explains condition to others	20	5	3.11	I was gonna use the same word. Exhausting, but almost because for me it’s something that is always, always on the back of my mind, and I’ve had experiences where you tell people, and then how much do you say, and it gets weird. I think I actually spend tons of energy pretending I’m totally fine and not telling anyone at all, and that’s just exhausting on a day-to-day basis.	1
Friendships	4	2	3.12	One of my bestest friends who I felt like I could talk to, like out of nowhere she’s like, “I just don’t think I can be your friend anymore and whenever I see you, you don’t seem happy to see me.” and I’m like “You’re the first person I was able to just be real with.	4
			3.13	I would say for me it’s been a huge struggle because if I start not feeling good or I’m not doing well I kinda isolate or, you know, and friends don’t understand why you kinda draw back or why you don’t always want to do certain things.	4
Coworkers	4	2	3.14	… They don’t treat me bad ’cause I’m a jerk at work, you know what I mean? Like, I’m a jerk and I let ’em know straight up, like, it’s not even your business but I’m telling you, and they know, they do things to help me out.	4
Adjustment to life with IC	11	4	3.15	Everything’s inconvenient. I just go to work and go home. That’s pretty much it. I make plans every once in a while but it’s hard to make plans, ’cause everything’s a big deal.	4
Quality of life					
Diet	47	6	3.16	I think it’s hard ’cause it’s not black and white. It’s some things work for others. It’s a lot of trial and error. And then it’s almost kind of like when you introduce food to a kid for the first time, you gotta do it one at a time so that [if you] do have a reaction you know what it is. It’s like you can cut this and you can cut that, but if you do too much at once, you don’t know what’s necessarily causing it to make it worse or better.	1
Financial	33	6	3.17	I’ve tried lots of different things. At one time I tried pelvic floor physical therapy. We tried to improve that, which wasn’t very successful, unfortunately. … All the cost associated with physical therapy, which is expensive, wasn’t worth the cost, unfortunately. That’s just my personal experience. It didn’t offer enough relief to justify the cost. …	2
Effect on work	25	6	3.18	It’s just really difficult. Not being able to go to work sometimes, that’s embarrassing. Not embarrassing, but it’s not like I can call my boss and be like, “Hey, I’m having a pelvic floor episode.” I don’t know, it’s just hard.	2
			3.19	I did the work thing for years. Worked the same job for the last 20 years. Loved it. Traveled. Had everything in the world that I could ever ask for in a job. But the last year that I was there, which is only three or four years now, I was in the hospital for a week five different times. What employer’s gonna keep you like that? It just didn’t work.	1
			3.2	The anxiety part. I used to not really … I’m not officially diagnosed with anxiety. I know that when you’re in a situation where you are, like at work, it’s embarrassing. Sometimes, I’m like blah. Share with everyone what’s going on. Depends who you are and how well I know you. There are just some days where I’m just like, I don’t know how I’m going to get to work and I start freaking out in my mind. Do I have to call my boss and tell them I can’t come in?	2
Travel	22	5	3.21	I just drove a car back from Nebraska, by the way, with my son. I drove it to Nebraska. He was most tolerant of me, because he knew that I was gonna have to stop at every exit. It was miserable.	1
			3.22	For me, like when I have a flare-up when it gets really bad, it’s like debilitating. … By the time I was done going to bathroom, getting back in the car, and he’d find a freeway entrance, I was like, “I have to go to the bathroom again.” I remember him handing me a cup and saying, “This is all in your head. I’ve got to get home.”	1
Planning activities/commitments	18	4	3.23	The inconvenience, I go to Disney World two summers ago, and after each ride I have to find a toilet, and my husband and sons. … We find a toilet, they get in line at the ride, and then I see where they’re in line. I go to the bathroom. And then I go catch up with them. It’s just inconvenient. You always have to know where a bathroom is.	1
Social isolation	14	4	3.24	I find it really lonely. I have to cook all my own meals. I can’t really do social things that my friends are going out to do. I can’t drink. I have to plan everything around where there’s a bathroom, where there’s food that I can eat, when can I go to bed, when can I take my meds. All of that becomes super isolating. …	1
			3.25	I push people away.	5
Provider capabilities					
Feels dismissed/not taken seriously	35	5	3.26	The neurologist that I first saw dismissed me. He didn’t believe in IC and straight up, “This is not real.” They put me in the hospital. I was in the hospital for about a week, because I couldn’t do anything. I was hurting so bad, but they never found anything that was causing it.	3
Misdiagnosis/alternative explanations	27	6	3.27	I was misdiagnosed like more times than I can possibly count. I was put on meds that made me so sick. They thought for a while that I had kidney problems, which I do, but they’re not necessarily related, or if they are, no one’s figured it out yet. I only recently was finally diagnosed, so it’s been like seven or eight years. … She diagnosed me just by looking through all of my other medical records, and said, “Well this is definitely what you have.” The medical world failed me for a long time, and it took a lot of fighting. Like, a lot of fighting. And telling people over and over and over, “No, you don’t understand. I’m in pain.”	1
Provider level of knowledge					
Sufficient	23	6	3.28	They gave me three shots of morphine in the hospital. Three and I was finally out. That’s how bad it was. The doctor actually came in, I was so lucky … He walked in the room and he told the nurses, he goes, “Give her morphine. My dad has IC.” He knew.	6
			3.29	Another thing, too, is, I was seeing two men. When I went to go see my gynecologist, which is a man, he said, “Why don’t you go see this female one? She’s been doing IC for 20 years. She’s very progressive, aggressive about her situations. Why don’t you go see her?”	5
Lacking	20	5	3.3	Try going to the emergency room with this. You gonna see a circus. It’s like a three-ring circus. “Why are you hurting?”, “Yes, I have kidney stones. I know I have kidney stones. I have bilateral, and there’s too many in there to count. I read the x-ray report just like you do. No, that’s not what’s hurting me.” “Yes, ma’am, I think it is.” “No, I’m telling you. I know the difference. It’s not.” All right, it’s a three-ring circus. Why can’t we go in and say, “I’m diagnosed with this. I need an emergency treatment.” They look at us like we’re crazy, or that you got horns growing, or you’re a druggie. And then you’ll have, on a chance, you’ll have a doctor that’ll say, “Yes, ma’am. You need an IV treatment. You just need fluids in there to get your body back in shape, ’cause you’ve dehydrated yourself. Let’s do that and let you rest for a little while.”	1
Delayed diagnosis	20	6	3.31	They didn’t diagnose mine for like 14 years.	4
			3.32	I don’t even have a set treatment yet. There is no treatment, but at this point, it’s like I feel like medicines are being thrown for symptoms that don’t seem to match up with what I’m saying. It’s just frustrating.	6
Feels she must advocate for herself	18	6	3.33	I love my doctors, but I’ve sworn off going to doctors anymore. I just absolutely had to because it’s hopeless. I’m sorry. It’s hopeless.	1
			3.34	I think I got really lucky and I got good doctors. I, at least, recently in the last, you know, maybe ten years, but you know, I am a big advocate. Everybody’s sick in my family, we have lots of doctors in my family and I’m not going to take no. And I’ll just go on and do my own research and talk to the doctors and try to find answers, but that’s because that’s not acceptable, whatever you’re telling me because that’s not true. But, like I was saying that many, many, many doctors are not educated. They’re just not. And I was really, really lucky when I first started I found a physician that only did women and only did IC. That’s it. So, I got really lucky. Right away.	4
Lack of communication between providers	5	3	3.35	Okay, everybody’s so specialized that they won’t talk to anybody else about it. Okay, when they gave me the Myrbetriq, my blood pressure went up. Well, I said, “Maybe I should take something for this, because I’m feeling real light headed and dizzy a lot.” The urologist said, “That’s not my job. I can’t tell you about your blood pressure.” I said, “Well, you’re the one that gave me the Myrbetriq that says, ‘Call your doctor or consult with your doctor if your blood pressure goes up,’ and mine’s gone up 30 points and I think we need to talk.” He said, “Talk?”	3

IC = interstitial cystitis; UTI = urinary tract infection; IV = intravenous.

Beyond romantic relationships, participants also detailed difficulties in family relationships, friendships, and relationships with coworkers as a result of symptoms. Participants reported a lack of understanding from family members and coworkers (quotations 3.09, 3.11) and experiencing the effects of disbelief and frustration by others (quotation 3.22). Relationship effects included socially isolating and withdrawing from friendships due to perceived inability of others to understand IC/BPS (quotation 3.13), diet restrictions (quotation 3.24), and excessive planning (quotation 3.15). Additional effects included expending significant energy on concealing symptoms from others (quotation 3.18) and friendships ending (quotation 3.12). Others reported job loss due to their condition (quotation 3.19).

In the larger social context, participants detailed burdens experienced as a result of navigating the health care system and interacting with a variety of providers to treat IC/BPS. Most participants described a delay in IC/BPS diagnosis (quotation 3.26) or misdiagnosis (quotation 3.22). Treatment experiences were characterized as highly variable and dependent upon finding providers specifically familiar with IC/BPS (quotation 3.29). Others noted experiencing disbelief and invalidation from physicians and a lack of physician familiarity with IC/BPS both inside urology and outside of physician subspecialty (quotations 3.21, 3.24, 3.25, 3.28), noting that this was a particular challenge when seeking emergent care (quotation 3.25).

In addition to the social context of IC/BPS, participants reported the numerous ways in which IC/BPS impacts daily life and decisions. This includes extensive planning of daily activities accounting for bathroom access (quotation 3.18), strict dietary regimens (quotation 3.19), and travel restrictions and inconveniences (quotations 3.16–3.18). Due to the stress and perceived burden on others, participants reported often staying at home as a result and experiencing loneliness and isolation (quotations 3.12, 3.19).

#### Theme 4: Response to Illness

(Table 4) Participants’ response to illness through methods of coping involved strategies considered both adaptive and maladaptive. Regarding adaptive coping, participants reported engaging in social support seeking (quotations 4.01, 4.05), self-advocacy, remaining socially active, using cognitive reframing (quotation 4.02), and seeking counseling to manage symptoms (quotations 4.10, 4.12). Others reported a noticeable lack of coping skills to manage pain and a lack of a support structure to assist with symptom management (quotation 4.09). Maladaptive coping strategies included excessive distraction, denial, symptom concealment, social isolation and withdrawal, and treatment noncompliance. Participants discussed seeking mental health support in five of six groups. Those who sought mental health services characterized their experiences as helpful, particularly in learning coping strategies to manage pain, although they noted that this as highly dependent on the provider. Participants also noted that mental health providers were often unfamiliar with the illness and not “knowledgeable” about IC/BPS or managing pain. One participant noted her counselor focusing on other issues outside of health and IC/BPS and dismissing her health complaints, stating that this was unhelpful. Others noted affordability as a primary barrier to seeking or continuing mental health services.

**Table 4.** Response to illness. Subthemes are sorted by number of mentions (decreasing) within each theme

Themes and subthemes	Mentions	Groups mentions (out of 6)	Number	Example quotation	Group
Social support/nonclinical coping strategies					
Self-care/other coping strategies	47	6	4.01	She’s very much all about the positive thinking, and I think that’s helped me a lot. A lot. I ask all my friends to try and be positive about it. Don’t ask me, “Oh, do you not feel well today?” No. I hate that. I hate being pitied. I hate being belittled about it. If I’m not laying in bed, let me try and be normal. Let me just try and do whatever it is that you’re doing, and if I can’t I’ll tell you.	1
			4.02	I’ve also started to appreciate more things around me because of it. That I am much more grateful with things I have, because of what I have been through or are going through. Just very grateful that I have kids or I have a house.	1
			4.03	I don’t think I cope very well with what I have going on. I can have pity parties. I’m not coping well with it. I think I just get really frustrated. Especially when I was diagnosed really young with an autoimmune disease, I just thought the only thing I can control is not taking medicine, which sounds really silly because I should be taking medicine. … I just don’t think I can.	2
			4.04	As far as coping skills, if I’m not having a good day, whether it’s the IC flare up and or really, really bad cramps. For period stuff, just a hint of cramping, I immediately take Aleve. A couple times I did this I didn’t mean to, but I took too much Aleve. I didn’t remember what the dosage was and I took two and then two later in the day or the evening. My mom was like, “What? You took how much? You’re not supposed to do that.” Then, also, last several months, even just one Aleve, if I’m taking it as the dosage is supposed to be, it doesn’t help the cramping. The cramping and the IC, it’s like both are happening at the same time. That’s really frustrating for me. I just want the pain to go away. I want to be able to function and not be doubled over in pain or wanting to lay in bed.	2
Family/friends/significant other	46	6	4.05	Overall, I’m lucky to have supportive people around me. I think I have pretty good coping skills just because I had a lot of childhood trauma growing up. I’ve learned how to overcome a lot of that. I’d say I’m pretty resilient in general. … The IC has been in control, but I’m afraid that if it doesn’t get better control that it will start to flare up.	2
			4.06	I take a lot of pain medication. I take medicine, Benadryl, I take something that’s a little bit stronger than that and try to survive on it, but that’s to go to sleep every night. If not, I’m … If I don’t get any sleep I’m just useless. You put somebody that’s sleep deprived on pain medication, and it’s like zonk, you’re out. You land in a corner somewhere. My children are grown. I don’t have the worry or concerns about the kids, but I do my grandchildren. They’re a big deal to me. I hate that for the longest my grandson would come in the house and say, “Aw, Nannie. We’re not gonna do anything again today?” And it would break my heart. It’d totally break my heart.	1
			4.07	My children are my survival line, if I need anything they are there. But I’ve been battling this since they were kids, so they understand that mom’s not good every day. That she has to run to the bathroom every 15 minutes on some occasions, and do not ask me to get in a car without a fight. I do not like to go anywhere. I just don’t like to travel anymore.	1
Support groups/online groups	16	5	4.08	I almost feel like structured group settings would be more beneficial. Then you have the piece of people that understand you but aren’t necessarily trained at moving the conversation. Someone that can reign you in and keep you from going down the rabbit hole. Like this, but not for a research study, more for a, I don’t know, once a week like a poker club or something.	6
No social support	8	4	4.09	I guess having the pain. I can handle the pain without let anybody know I’m in pain. … I don’t say anything. It would be good to have someone I could talk to about my problems.	2
Sought mental health support					
Yes, has sought mental health support	27	5	4.1	I have seen a couple therapists over the years. Just recently I have started going back to a guy that’s really, I really clicked with him, and it’s not been long enough for me to. … I’m still working through a lot of stuff, talking with him, as I’ve only seen him a couple times in the last couple of weeks, but I do feel better after I talk about it with someone who can interpret my what I call gibberish. I feel like I can’t speak about what I’m feeling, I can’t put it into words, but he’s able to tell me what he hears me say, so I can understand it better, and I feel that helps me try to deal with this life and in turn that kinda helps me, gives me a little more confidence that I can continue to find things that help me.	3
			4.11	Well, I mean mine all came about through like a—I was seeing somebody and I think it was the—of course it takes a lot of times to find the right person who’s the right fit and as soon as I did, I mean, they’ve been very great about, but once again, I can’t pay a hundred and fifty to go see my therapist, you know? So that’s kinda the hard part.	4
Level of effectiveness of mental health support	16	5	4.12	But it was great to have finally a doctor who—a psychologist who actually understood it and so a lot of things were geared toward figuring out ways to cope with things, that did make a big difference.	4
			4.13	It’s been really helpful, yeah. But it’s not even something I thought to do until I came to the Osher Center, and one of the MDs said, “We have this person, so would you be interested?” I didn’t even think that that would be something I might need to do until I tried it.	1
Number of visits necessary	12	4	4.14	I mean, I think the ideal scenario is when you’re first diagnosed, a year. To really help ’cause during that year they’re going to try different medications, they’re going to try bladder installations, I mean, they’re going to try a bunch of different stuff and not everything works for everybody and somethings irritate people more than, you know, others, so having that constant support until you really do have a better idea of it would be. … I’ve never had that opportunity to really have that constant support or talking through it or having somebody who really understands.	4
			4.15	I guess if I felt like it was a high stress period, like more stuff going on and I felt like I was having more of a flare up period, I would probably want to see that person more frequently. Maybe once a week, if needed, twice a week. I would imagine, for me at this point, if I were to see someone once every two or three weeks, maybe.	2
			4.16	Or yeah, I guess, lifelong.	4
No, has not sought mental health support	3	3	4.17	… It’s almost like a team effort, things are better. I personally hadn’t tried the therapy, but I come from a mental health background, so I think part of that is just it’s hard to go see a therapist when that’s your background. It’s just been hard for me. And then but I think it … What’s the saying? It takes a village.	1

IC = interstitial cystitis.

#### Theme 5: Addressing Needs in Treatment

(Table 5) Participants described mixed experiences with treatment and variable degrees of satisfaction with outcomes. Most characterized their treatment experiences as unsuccessful based on a trial-and-error approach to treatment. Participants expressed a desire for increased awareness, research, and provider education in IC/BPS (quotations 5.01, 5.11). Further, participants expressed a need for patient education materials on IC/BPS distributed from a reputable, trustworthy source (quotations 5.04, 5.07). Specifically, participants reported self-educating through seeking out information online and not knowing its reliability or accuracy. Participants questioned provider capabilities and knowledge of IC/BPS. Others expressed a lack of knowledge about IC/BPS physiology, treatment options, and self-management tools to use adjunctive to medical treatments (quotations 5.04, 5.07).

**Table 5.** Perceived needs in treatment. Subthemes are sorted by number of mentions (decreasing)

Themes and subthemes	Mentions	Group mentions (out of 6)	Number	Example quotation	Group
Perceived needs in treatment					
What do doctors need to know	44	4	5.01	Well, let’s just first start with informing our lay people out there and your regular doctor doctor, your primary care doctor, your regular urologist. They don’t believe it exists. It’s kinda like, you know, women didn’t have cramps or … it’s asinine that urology doctors aren’t informed. It’s asinine. They need to be educated. They need to get the word out.	4
Important factors for treatment	37	6	5.02	I think it’d be helpful to have a support group, just to be able to have other people’s experiences and what they’ve tried, what hasn’t worked, what does work, but also a counselor.	2
Support groups	27	4	5.03	It kills me because there’s no … and I’ve thought about doing this, too. This is great that I’m glad we’re here is that my psychologist that I’ve worked with … emotional-wise has been great to talk to, but they don’t have it so they can’t really understand it. I think when you work with somebody that actually has it and you can talk to somebody, the three of us could sit in here and probably … We could probably not stop talking for 24 hours straight because of all the stuff we have in common.	6
Adequate treatment	21	4	5.04	Even if there was a little book or I don’t know. I feel like you can go to the internet and there are so many different websites and everyone tells you something different. …	4
			5.05	I would say lots of tests. For me, the only reason why I knew I had go 62 times a day is because I had to do a tally mark.	5
			5.06	In general, I haven’t really gotten into it too much, or been concerned with it, just ’cause there’s a lot of other stuff that I’m still juggling. … Also, whenever I do read about it or educate myself, I kind of just freak out a little bit. It’s just so overwhelming and just so negative for me that it’s just easy to like, “Okay, I’m just gonna keep doing what I’m doing.” And just be open to more things like this [group], because I really do feel like this is helpful and in the right direction.	1
Needs better personal understanding of IC	15	3	5.07	That’s another thing. They say that when you have multiple children your bladder gets weaker. Your bladder does this. Your bladder does that. Again, what is my IC doing to me? That’s what I can never find out.	5
			5.08	I thought something was horribly wrong and it was nothing in my urine. It was like I was still in those stages like you like, “What is wrong with me?” Oh, you have a bacterial infection and you have IC. It was not having all the tools to know what’s wrong with you all the time can make somebody so crazy and make you feel like you’re out of whack all the time.	6
Symptom management	13	5	5.09	Talking to counselors is probably the number one thing for me right now that’s missing. You know, for me, stress being such a flare for me that having somebody to talk to professionally I think would really help.	4
Day-to-day IC management needs	11	4	5.10	I think a pain medication that’s not addicting. I don’t want to take pain medication because it’s so addicting, but I just want to be out of pain. I just wish there was something that you could do, that you could take that would work and not be addicting.	2
			5.11	Well, I think if they would define it. If they would say, “This is what it is and this is what you can do.” The dye, instead of just throwing the medication, if they say … I have not been told anything that I can do personally to relieve it. And that really bothers me.	3

IC = interstitial cystitis.

Regarding symptom management, participants reported a desire for mental health services integrated into a collaborative care model addressing both the physiological and psychological aspects of the condition in tandem. Some expressed a desire for structured support groups involving other IC/BPS patients in order to share knowledge, resources, and experiences (quotations 5.02, 5.03). For managing day-to-day symptoms, participants expressed a desire for nonaddictive methods of managing pain, alternative treatments, and specific instruction in tools to use independently to manage symptoms at home (quotation 5.10). Regarding psychological treatment, participants reported a desire for individual sessions in order to get to specifics influencing triggers and a “customized” approach to pain management, particularly during flare periods. Importantly, participants expressed a desire for an individualized care plan, recognizing that others have mixed symptom constellations and triggers that may warrant different treatment approaches.

### Self-report of Symptoms

Clinically (Table 1), the overall sample reported moderate-to-severe IC/BPS symptoms^29^ (*M*_ICSI_ = 12.44, SD = 4.80, Cronbach’s α = 0.85), moderate pain levels, and a high degree of widespread pain (*M*_MBM_ = 10.52 pain sites, SD = 11.01, Cronbach’s α = 0.97). Participants also indicated mild levels of depression symptoms^30^ (*M*_PHQ-9_ = 8.46, SD = 7.37, Cronbach’s α = 0.96) and 14% of individuals (*N* = 4/27) reported some degree of suicidal ideation on the PHQ-9 (item 9). Levels of anxiety fell nearly one standard deviations above the mean of the general population (*M*_PROMIS_ = 20.77, SD = 8.29, *t*-score = 59.0, Cronbach’s α = 0.95).^14^ Both anxiety and depression symptoms were correlated with overall severity of IC/BPS, *r*_PROMIS_(24) = 0.48, *p* = 0.013; *r*_PHQ-9_(24) = 0.68, *p* < 0.001. The full data were unavailable for analyses involving depression and anxiety symptoms. One participant was missing an item on the PROMIS anxiety scale and a different participant was missing an item on the PHQ-9 scale, so these participants were excluded from reliability analyses and the following analyses. Overall, our sample’s symptom characteristics resemble those of other larger clinical and community cohort studies,^6,7,23^ with one exception. It appears that, on average, our sample had higher degrees of widespread pain.^42^

We first examined the differential predictive value of anxiety and depression on IC/BPS symptoms to assess whether the quantitative findings would converge with qualitative themes. In a multiple regression model with both depression and anxiety symptoms predicting severity of IC/BPS as measured by total ICSI/ICPI score, only depression symptoms was a significant predictor (β = 0.66, SE = 0.30, *t* = 2.58, *p*= 0.02, *R*^2^ = 0.43). Though depression and anxiety symptom measures were correlated, *r*(23) = 0.78, *p* < 0.001, there was no evidence of multicollinearity in the model, variance inflation factors (VIFs) = 2.56. VIFs > 5 are generally considered problematic (see, e.g., James et al.^43^). It appeared that this model converged with the qualitative findings regarding role of psychological factors in IC/BPS. Depression symptoms appeared to better capture the role of psychological factors better than anxiety symptoms.

Given that depression symptoms were more predictive of IC/BPS symptoms, we then assessed the confounding factors of age and time since diagnosis, both of which have been related to increased symptom and depression symptom severity in previous investigations. . When accounting for age and time since diagnosis (in years), depression symptoms significantly predicted IC/BPS severity (β = 0.67, SE = 0.19, *t* = 4.08, *p* < 0.001, *R*^2^ = 0.46; VIFs < 3.50). Depression symptoms appeared relavant to IC/BPS severity independent from the effects of age and the duration of IC/BPS diagnosis.

## Discussion

Study findings confirmed the significant psychosocial burden accompanying IC/BPS. In our sample, consistent with previous investigations, depression symptoms were significantly associated with increased IC/BPS symptom severity, irrespective of age or symptom duration. Qualitative analyses reiterated the reciprocal relationship between stress and urologic symptoms. Further, patients emphasized persistent preoccupation and worry about urinary frequency, urgency, and bladder pain, placing significant cognitive effort into both accommodating symptoms and preparing for anticipated symptom exacerbations.

Qualitative reports echoed known disruptions of IC/BPS on daily life while providing a detailed account of interpersonal struggles stemming from IC/BPS, with romantic relationships being a particular challenge. Participants voiced fear of sexual activity and associated pain ending existing relationships and leading to avoidance of dating. Many participants reported experiencing misunderstanding and invalidation from loved ones and difficulty communicating needs to partners, family members, and coworkers. Participants reported social withdrawal and isolation, concealment of symptoms, and avoidance of others/activity as a result. To improve IC/BPS management, participants expressed a desire for a multimodal approach to treatment with regular collaboration between providers, improved education on IC/BPS, nonaddictive pain management strategies, and a need to integrate psychological care with their medical care.

### Biopsychosocial Framework Applied to IC/BPS

**Figure 2.**
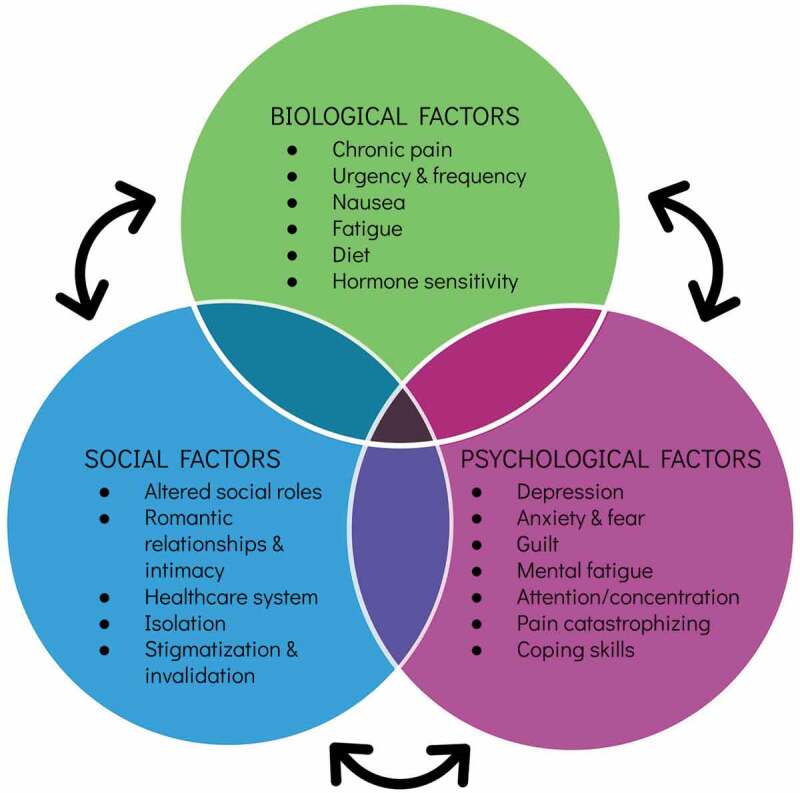
Biopsychosocial model of IC/BPS

The conceptual framework derived from our qualitative analysis closely resembles biopsychosocial model of pain that accounts for the impact of a disease (i.e., IC/BPS) as filtered through an individual’s genetics, learning history, pain modulation, psychological status, expectations, and sociocultural environment, reflecting the expression of a person’s *illness*.^44^ Thus, the complex interaction of individual biological, psychological, and social factors influences how a person perceives, responds to, and copes with an illness.^44,45^ Importantly, the relative influence of each factor differs for the individual, varies throughout the course of illness, and may shift over time. Figure 2 indicates the biopsychosocial model as adapted to IC/BPS, with content themes inductively derived from this investigation. This model encompasses both a conceptualization of the patient experience and a desired treatment framework by patients. Consistent with a recent investigation examining the impact of IC/BPS,^35^ study findings reinforce that patients strongly crave a collaborative, personalized approach to IC/BPS treatment addressing symptoms, their emotional impact, and interference with major life domains. Participants acknowledged variable treatment experiences with providers and managing IC/BPS. They expressed frustration with a trial-and-error treatment approach. The majority of participants characterized their medical treatment as unsuccessful overall. Use of the medical model with IC/BPS, which focuses intently on biology and physiological symptoms, may bypass crucial psychosocial treatment targets,^46^ lead to ineffective treatment, and breed mistrust in patient–provider relationships.

### Treatment Implications

Recent investigations acknowledge the lack of interdisciplinary mental health intervention in urology, despite the mounting evidence of the psychosocial difficulties that accompany urologic disease and guidelines for their management.^15,47^ Our study findings, preliminary evidence from pilot trials, and research in associated conditions (e.g., irritable bowel syndrome^48^) suggest that patients may benefit from a biopsychosocial model of care. Recent calls to action from national societies reflect a similar sentiment from providers managing benign urologic disease.^49^ Therefore, an optimal treatment approach could address physiological contributors to IC/BPS and simultaneously attune to patient cognitions, emotions, and behaviors that may impact symptoms and functional status. Regarding pain, psychological and environmental factors modulate nociception and treatment response and vice versa. Psychosocial intervention can provide patients with techniques to gain a sense of control over the effects of pain on their lives by modifying the affective, behavioral, cognitive, and sensory facets of the experience. Interventions may also help address the interpersonal impacts of IC/BPS. For IC/BPS, psychosocial intervention could be delivered adjunctively via therapist referral or intergratively though embedding psychological providers in-clinic. Integrative subspecialty clinics have performed successfully in associated settings, with patients having less clinic utilization and proceudres after an average of four sessions with a psychologist.^50^ This is financially feasible with the use of health and behavior codes, designed specifically for health psychologists to address the influence of stress on medical conditions in medically based visits.

To our knowledge, three small pilot investigations to date have examined some form of psychological intervention for IC/BPS, including skills delivery individually, in groups, and online.^51–53^ Though there were indications of symptom improvement, particularly in those receiving guided imagery, these interventions had small sample sizes and variable treatment effects with limited follow-up.

To advance existing cognitive–behavioral treatments for pain, leading researchers call for illness-specific interventions matched to patient needs.^19^ Specifically, the “one-size-fits-all” approach may not maximize the benefits of psychological treatment for different pain conditions. How the form and content of pain shapes patient experience is largely unexplored.^19^ For example, the location of pain—say, in the pelvis and urethra—may carry an entirely different meaning or implication than neuropathy in the extremities. It is clear that IC/BPS-related pain and sexual dysfunction impact patient behavior and relationships. This finding mirrors qualitative inquiries of vulvodynia where patients identified shame, guilt, communication difficulties, and relationship strain due to dyspareunia.^54,55^ There are initial indications of self-management interventions improving IC/BPS symptoms and quality of life.^15,51,53^ Our study provides additional insight into specific elements of focus for psychosocial intervention. An intervention that provides pain coping skills and also addresses assertive communication, fear and avoidance of relationships and intimacy, and sexual dysfunction would align with patient needs expressed in this investigation. Replacing fear and rumination with adaptive coping would also be beneficial. In addition, targeting depression symptoms could lead to further symptom reduction. Patients desire education and skills building for IC/BPS management, which can be supported by cognitive–behavioral approaches to pain with specific education. To address the pervasive relationship dysfunction with loved ones, friends, colleagues, and medical providers due to IC/BPS we suggest incorporating interpersonal principles and assertiveness training in treatment when possible.^56^ Specifically, interpersonal therapy identifies role transitions and disputes in relationships that contribute to distress (for IC/BPS examples, see Table 3), working supportively to enhance the patient’s ability to assert needs in interpersonal encounters. Therefore, an optimal psychological intervention could include illness-specific education and integrate interpersonal^56^ principles with cognitive–behavioral^18^ methodologies to address the pain, depression, and significant relationship dysfunction that arise as a result of living with IC/BPS.

### Study Limitations

Our study is cross-sectional, limiting our ability to draw causal conclusions from data. Our method of recruitment may have led to some sampling bias due to requiring in-person attendance. In some cases, those reporting high pain states with a longer distance to drive were less likely to attend focus groups (e.g., two participants confirmed but did not attend reportedly due to pain and fear that driving would exacerbate pain). Because higher pain levels have been connected to increased psychological distress, our quantitative data may have underestimated these values in our sample. No participants were excluded on the basis of active suicidal ideation at the time of screening; this advertised study exclusion may have deterred highly depressed individuals from attempting to enroll. However, our sample’s level of symptom severity, psychological distress, suicidality, and anxiety are comparable to data collected in much larger clinical and community cohorts. We experienced variability in group size, which ranged from 2 to 12 participants. With fewer individuals present, group process may evolve differently, providing more opportunity for in-depth discussion; however, the breadth of discussion and frequency of voiced concerns may have been affected in smaller cohorts. Qualitative analyses tend to have smaller sample sizes, because thematic saturation can be reached with fewer than 30 group participants.^57^ This limits our quantitative analysis power due to a relatively low sample size. Despite recruiting within a medical center and surrounding communities, our study included only females with IC/BPS, of whom nearly half were working full time and may have been of relatively higher socioeconomic status than may be found in other populations. Sample homogenity is a common limitation to generalizability in the available research on IC/BPS. Existing studies lack racial and socioeconomic diversity, with data collected primarily in outpatient specialty clinics, where patients have access to care and health insurance.^10^ Different findings might emerge from studying a group that included men or individuals with different levels of function and financial resources. Lastly, we were missing clinical diagnostic information from three participants. We used validated cutoff scores consistent with a diagnosis of IC/BPS^29^ gathered in pre-group assessments as a diagnostic surrogate. Previous investigations applied similar methods when using epidemiological criteria to evaluate symptom presentations between clinically diagnosed and community cohorts of women females with IC/BPS, finding comparable clinical presentations between groups.^12^

## Conclusion

Patients with IC/BPS have significant unment psychosocial needs, particularly in addressing sexual and relationship dysfunction. In adapting psychosocial intervention to this population, tailoring existing cognitive–behavioral interventions for pain to IC/BPS by addressing the depression, educational needs for the condition, and significant relationship and sexual dysfunction associated with IC/BPS will likely best meet patient expressed needs. Further research is needed to formally test the benefits of a patient-informed cognitive–behavioral intervention for IC/BPS in a randomized, adequately powered trial that assesses treatment benefits for all genders.

## References

[1] Berry SH, Elliott MN, Suttorp M, Bogart LM, Stoto MA, Eggers P, Nyberg L, Clemens JQ. Prevalence of symptoms of bladder pain syndrome/interstitial cystitis among adult females in the United States. J Urol. 2011;186(2):540–44. doi:10.1016/j.juro.2011.03.132. 21683389 PMC3513327

[2] Payne CK, Joyce GF, Wise M, Clemens JQ. Interstitial cystitis and painful bladder syndrome. J Urol. 2007;177(6):2042–49. doi:10.1016/j.juro.2007.01.124. 17509284

[3] Hanno PM, Erickson D, Moldwin R, Faraday MM. Diagnosis and treatment of interstitial cystitis/bladder pain syndrome: AUA guideline amendment. J Urol. 2015;193(5):1545–53. doi:10.1016/j.juro.2015.01.086. 25623737

[4] Blumenthal D, Chernof B, Fulmer T, Lumpkin J, Selberg J. Caring for high-need, high-cost patients — an urgent priority. N Engl J Med. 2016;375(10):909–11. doi:10.1056/NEJMp1608511. 27602661

[5] Hanno PM, Burks DA, Clemens JQ, Dmochowski RR, Erickson D, Fitzgerald MP, Forrest JB, Gordon B, Gray M, Mayer RD, et al. AUA guideline for the diagnosis and treatment of interstitial cystitis/bladder pain syndrome. J Urol. 2011;185(6):2162–70. doi:10.1016/j.juro.2011.03.064. 21497847 PMC9341322

[6] Watkins KE, Eberhart N, Hilton L, Suttorp MJ, Hepner KA, Clemens JQ, Berry SH. Depressive disorders and panic attacks in women with bladder pain syndrome/interstitial cystitis: a population-based sample. Gen Hosp Psychiatry. 2011;33(2):143–49. doi:10.1016/j.genhosppsych.2011.01.004. 21596207 PMC3099040

[7] Hepner KA, Watkins KE, Elliott MN, Clemens JQ, Hilton LG, Berry SH. Suicidal ideation among patients with bladder pain syndrome/interstitial cystitis. Urology. 2012;80(2):280–85. doi:10.1016/j.urology.2011.12.053. 22658505 PMC3411912

[8] Tripp DA, Nickel JC, Krsmanovic A, Pontari M, Moldwin R, Mayer R, Carr LK, Yang CC, Nordling J. Depression and catastrophizing predict suicidal ideation in tertiary care patients with interstitial cystitis/bladder pain syndrome. Can Urol Assoc. 2016;10(11–12):383–88. doi:10.5489/cuaj.3892. PMC5167592 28096911

[9] Goldstein HB, Safaeian P, Garrod K, Finamore PS, Kellogg-Spadt S, Whitmore KE. Depression, abuse and its relationship to interstitial cystitis. Int Urogynecol J. 2008;19(12):1683–86. doi:10.1007/s00192-008-0712-x. 18766291

[10] Cox A, Golda N, Nadeau G, Nickel JC, Carr L, Corcos J, Teichman J. CUA guideline: diagnosis and treatment of interstitial cystitis/bladder pain syndrome. Can Urol Assoc. 2016;10(5–6):E136. doi:10.5489/cuaj.3786. PMC5065402 27790294

[11] Chuang Y-C, Weng S-F, Hsu Y-W, Huang -CL-C, Wu M-P. Increased risks of healthcare-seeking behaviors of anxiety, depression and insomnia among patients with bladder pain syndrome/interstitial cystitis: a nationwide population-based study. Int Urol Nephrol. 2015;47(2):275–81. doi:10.1007/s11255-014-0908-6. 25577231

[12] Konkle KS, Berry SH, Elliott MN, Hilton L, Suttorp MJ, Clauw DJ, Clemens JQ. Comparison of an interstitial cystitis/bladder pain syndrome clinical cohort with symptomatic community women from the RAND Interstitial Cystitis Epidemiology study. J Urol. 2012;187(2):508–12. doi:10.1016/j.juro.2011.10.040. 22177158 PMC3894739

[13] Clemens JQ, Clauw DJ, Kreder K, Krieger JN, Kusek JW, Lai H, Rodriguez L, Williams DA, Xiaoling H, Stephens A, et al. Comparison of baseline urologic symptoms in men and women in the multidisciplinary approach to the study of chronic pelvic pain (MAPP) research cohorT. J Urol. 2015;193(5):1554–58. doi:10.1016/j.juro.2014.11.016. 25463989 PMC4454891

[14] Pilkonis PA, Choi SW, Reise SP, Stover AM, Riley WT, Cella D. Item banks for measuring emotional distress from the Patient-Reported Outcomes Measurement Information System (PROMIS®): depression, anxiety, and anger. Assessment. 2011;18(3):263–83. doi:10.1177/1073191111411667. 21697139 PMC3153635

[15] McKernan LC, Walsh CG, Reynolds WS, Crofford LJ, Dmochowski RR, Williams DA. Psychosocial co-morbidities in Interstitial Cystitis/Bladder Pain syndrome (IC/BPS): a systematic review. Neurourol Urodyn. 2017;37(3):926–941. 10.1002/nau.23421 PMC6040587 28990698

[16] Koh JS, Ko HJ, Wang SM, Cho KJ, Kim JC, Lee S-J, Pae C-U. The impact of depression and somatic symptoms on treatment outcomes in patients with chronic prostatitis/chronic pelvic pain syndrome: a preliminary study in a naturalistic treatment setting. Int J Clin Pract. 2014;68(4):478–85. doi:10.1111/ijcp.12340. 24471930

[17] Lee SWH, Liong ML, Yuen KH, Leong WS, Cheah PY, Khan NAK, Krieger JN. Adverse impact of sexual dysfunction in chronic prostatitis/chronic pelvic pain syndrome. Urology. 2008;71(1):79–84. doi:10.1016/j.urology.2007.08.043. 18242370

[18] Williams DA, In DJ, Philadelphia PA. Cognitive and behavioral approaches to chronic pain. In: Clauw WDJ editor. Fibromyalgia and other central pain syndromes. Philadelphia (PA): Lippincott Williams Wilkins; 2005. p. 343–52.

[19] Eccleston C, Crombez G. Advancing psychological therapies for chronic pain. F1000Research. 2017;6:461–461. doi:10.12688/f1000research.10612.1. 28413627 PMC5389407

[20] Harris PA, Scott KW, Lebo L, Hassan N, Lighter C, Pulley J. ResearchMatch: a national registry to recruit volunteers for clinical research. Acad Med. 2012;87(1):66. doi:10.1097/ACM.0b013e31823ab7d2. 22104055 PMC3688834

[21] Dworkin RH, Turk DC, Farrar JT, Haythornthwaite JA, Jensen MP, Katz NP, Kerns RD, Stucki G, Allen RR, Bellamy N, et al. Core outcome measures for chronic pain clinical trials: IMMPACT recommendations. Pain. 2005;113(1–2):9–19. doi:10.1016/j.pain.2004.09.012. 15621359

[22] Williams DA. The importance of psychological assessment in chronic pain. Curr Opin Urol. 2013;23(6):554–59. doi:10.1097/MOU.0b013e3283652af1. 24080806 PMC4295636

[23] Landis JR, Williams DA, Lucia MS, Clauw DJ, Naliboff BD, Robinson NA, van Bokhoven A, Sutcliffe S, Schaeffer AJ, Rodriguez LV, et al. The MAPP research network: design, patient characterization and operations. BMC Urol. 2014;14(1):1. doi:10.1186/1471-2490-14-58. 25085119 PMC4126395

[24] Keller S, Bann CM, Dodd SL, Schein J, Mendoza TR, Cleeland CS. Validity of the brief pain inventory for use in documenting the outcomes of patients with noncancer pain. Clin J Pain. 2004;20(5):309–18. doi:10.1097/00002508-200409000-00005. 15322437

[25] Brummett CM, Hassett AL, Brummett KA, Clauw DJ, Williams DA. The Michigan Body Map and its use in assessing the American College of Rheumatology survey criteria for Fibromyalgia. Arthritis Rheum. 2011;62:744.

[26] Brummett CM, Bakshi RR, Goesling J, Leung D, Moser SE, Zollars JW, Williams DA, Clauw DJ, Hassett AL. Preliminary validation of the Michigan Body Map (MBM). Pain. 2016;157(6):1205–12. doi:10.1097/j.pain.0000000000000506. 26835782 PMC4868633

[27] Tong A, Sainsbury P, Craig J. Consolidated criteria for reporting qualitative research (COREQ): a 32-item checklist for interviews and focus groups. Int J Qual Health Care. 2007;19(6):349–57. doi:10.1093/intqhc/mzm042. 17872937

[28] O’Leary MP, Sant GR, Fowler FJ, Whitmore KE, Spolarich-Kroll J. The interstitial cystitis symptom index and problem index. Urology. 1997;49(5):58–63. doi:10.1016/S0090-4295(99)80333-1. 9146003

[29] Clemons JL, Arya LA, Myers DL. Diagnosing interstitial cystitis in women with chronic pelvic pain. Obstet Gynecol. 2002;100(2):337–41. doi:10.1016/s0029-7844(02)02087-2. 12151160

[30] Kroenke K, Spitzer RL, Williams JBW. The PHQ-9: validity of a brief depression severity measure. J Gen Intern Med. 2001;16(9):606–13. doi:10.1046/j.1525-1497.2001.016009606.x. 11556941 PMC1495268

[31] Gilbody S, Richards D, Brealey S, Hewitt C. Screening for depression in medical settings with the Patient Health Questionnaire (PHQ): a diagnostic meta-analysis. J Gen Intern Med. 2007;22(11):1596–602. doi:10.1007/s11606-007-0333-y. 17874169 PMC2219806

[32] Amtmann D, Cook KF, Jensen MP, Chen WH, Choi S, Revicki D, Cella D, Rothrock N, Keefe F, Callahan L, et al. Development of a PROMIS item bank to measure pain interference. Pain. 2010;150(1):173–82. doi:10.1016/j.pain.2010.04.025. 20554116 PMC2916053

[33] Cella D, Yount S, Rothrock N, Gershon R, Cook K, Reeve B, Ader D, Fries JF, Bruce B, Rose M. The Patient-Reported Outcomes Measurement Information System (PROMIS): progress of an NIH roadmap cooperative group during its first two years. Med Care. 2007;45(5 Suppl 1):S3–S11. doi:10.1097/01.mlr.0000258615.42478.55. PMC2829758 17443116

[34] *IBM SPSS statistics for windows, version 22* [computer program]. Armonk (NY): IBM Corp; 2013. https://www.ibm.com/support/pages/how-cite-ibm-spss-statistics-or-earlier-versions-spss

[35] Nickel JC, Tripp DA, Beiko D, Tolls V, Herschorn S, Carr LK, Kelly K-L, Golda N. The interstitial cystitis/bladder pain syndrome clinical picture: a perspective from patient life experience. Urol Pract. 2018;5(4):286–92. doi:10.1016/j.urpr.2017.06.005. 37312294

[36] Saunders B, Sim J, Kingstone T, Baker S, Waterfield J, Bartlam B, Burroughs H, Jinks C. Saturation in qualitative research: exploring its conceptualization and operationalization. Qual Quant. 2018;52(4):1893–907. doi:10.1007/s11135-017-0574-8. 29937585 PMC5993836

[37] Maxwell J. Understanding and validity in qualitative research. Harv Educ Rev. 1992;62(3):279–301. doi:10.17763/haer.62.3.8323320856251826.

[38] Fereday J, Muir-Cochrane E. Demonstrating rigor using thematic analysis: a hybrid approach of inductive and deductive coding and theme development. Int. J. Qual. Methods. 2006;5(1):80–92. doi:10.1177/160940690600500107.

[39] Turk DC, Monarch ES. Biopsychosocial perspective on chronic pain. In: Turk DC, Gatchel RJ editors. Psychological approaches to pain management. Vol. 2. New York (NY): Guilford Press; 2002. p. 3–30.

[40] Novi JM, Jeronis S, Srinivas S, Srinivasan R, Morgan MA, Arya LA. Risk of irritable bowel syndrome and depression in women with interstitial cystitis: a case-control study. J Urol. 2005;174(3):937–40. doi:10.1097/01.ju.0000169258.31345.5d. 16093997

[41] Rothrock NE, Lutgendorf SK, Hoffman A, Kreder KJ. Depressive symptoms and quality of life in patients with interstitial cystitis. J Urol. 2002;167(4):1763–67. doi:10.1016/S0022-5347(05)65195-6. 11912405

[42] Naliboff BD, Stephens AJ, Afari N, Lai H, Krieger JN, Hong B, Lutgendorf S, Strachan E, Williams D. Widespread psychosocial difficulties in men and women with urologic chronic pelvic pain syndromes: case-control findings from the multidisciplinary approach to the study of chronic pelvic pain research network. Urology. 2015;85(6):1319–27. doi:10.1016/j.urology.2015.02.047. 26099876 PMC4479402

[43] James G, Witten D, Hastie T, Tibshirani R. An introduction to statistical learning. Vol. 112. Springer; 2013.

[44] Turk DC, Monarch ES. Biopsychosocial perspective on chronic pain. In: Turk DC, Gatchel RJ editors. Psychological approaches to pain management: a practitioner’s handbook. 3rd ed. New York (NY): The Guilford Press; 2018. p. 3–24.

[45] Gatchel RJ, Peng YB, Peters ML, Fuchs PN, Turk DC. The biopsychosocial approach to chronic pain: scientific advances and future directions. Psychol Bull. 2007;133(4):581. doi:10.1037/0033-2909.133.4.581. 17592957

[46] Bosch PC, Bosch DC. Treating interstitial cystitis/bladder pain syndrome as a chronic disease. Rev Urol. 2014;16:83–87. 25009448 PMC4080853

[47] Kinsey D, Pretorius S, Glover L, Alexander T. The psychological impact of overactive bladder: a systematic review. J Health Psychol. 2016;21(1):69–81. doi:10.1177/1359105314522084. 24591118

[48] Laird KT, Tanner-Smith EE, Russell AC, Hollon SD, Walker LS. Comparative efficacy of psychological therapies for improving mental health and daily functioning in irritable bowel syndrome: a systematic review and meta-analysis. Clin Psychol Rev. 2017;51:142–52. doi:10.1016/j.cpr.2016.11.001. 27870997

[49] von Gontard A, Vrijens D, Selai C, Mosiello G, Panicker J, von Koeveringe G, Apostolidis A, Anding R. Are psychological comorbidities important in the aetiology of lower urinary tract dysfunction—ICI‐RS 2018? Neurourol Urodyn. 2019;38:S8–S17. doi:10.1002/nau.24016. 31059602

[50] Kinsinger SW, Ballou S, Keefer L. Snapshot of an integrated psychosocial gastroenterology service. World J Gastroenterol. 2015;21(6):1893–99. doi:10.3748/wjg.v21.i6.1893. 25684957 PMC4323468

[51] Carrico DJ, Peters KM, Diokno AC. Guided imagery for women with interstitial cystitis: results of a prospective, randomized controlled pilot study. J Altern Complement Med. 2008;14(1):53–60. doi:10.1089/acm.2007.7070. 18199015

[52] Lee MH, Wu HC, Lin JY, Tan TH, Chan PC, Chen YF. Development and evaluation of an E-health system to care for patients with bladder pain syndrome/interstitial cystitis. Int J Urol. 2014;21(Suppl 1):62–68. doi:10.1111/iju.12336. 24807502

[53] Kanter G, Komesu YM, Qaedan F, Jeppson PC, Dunivan GC, Cichowski SB, Rogers RG. Mindfulness-based stress reduction as a novel treatment for interstitial cystitis/bladder pain syndrome: a randomized controlled trial. Int Urogynecol J. 2016;27(11):1705–11. doi:10.1007/s00192-016-3022-8. 27116196 PMC5067184

[54] Sadownik LA, Smith KB, Hui A, Brotto LA. The impact of a woman’s dyspareunia and its treatment on her intimate partner: a qualitative analysis. J Sex Marital Ther. 2017;43(6):529–42. doi:10.1080/0092623X.2016.1208697. 27398766

[55] Shallcross R, Dickson JM, Nunns D, Mackenzie C, Kiemle G. Women’s subjective experiences of living with vulvodynia: a systematic review and meta-ethnography. Arch Sex Behav. 2018;47(3):577–95. doi:10.1007/s10508-017-1026-1. 28905128 PMC5834572

[56] Markowitz JC, Weissman MM. Interpersonal psychotherapy: principles and applications. World Psychiatry. 2004;3:136–39. 16633477 PMC1414693

[57] Guest G, Bunce A, Johnson L. How many interviews are enough? An experiment with data saturation and variability. Field Methods. 2006;18(1):59–82. doi:10.1177/1525822X05279903.

